# Enhancing the Efficacy of Chloramphenicol Therapy for *Escherichia coli* by Targeting the Secondary Resistome

**DOI:** 10.3390/antibiotics13010073

**Published:** 2024-01-12

**Authors:** Mosaed Saleh A. Alobaidallah, Vanesa García, Sandra M. Wellner, Line E. Thomsen, Ana Herrero-Fresno, John Elmerdahl Olsen

**Affiliations:** 1 Department of Veterinary and Animal Sciences, Faculty of Health and Medical Sciences, University of Copenhagen, 1870 Frederiksberg, Denmark; obaidallahm@ksau-hs.edu.sa (M.S.A.A.); vanesag.menendez@usc.es (V.G.); sandra.wellner@sund.ku.dk (S.M.W.); leth@sund.ku.dk (L.E.T.); 2 Department of Clinical Laboratory Sciences, College of Applied Medical Sciences, King Saud bin Abdulaziz University for Health Sciences, Jeddah 21423, Saudi Arabia; 3 King Abdullah International Medical Research Center, Jeddah 22384, Saudi Arabia; 4 Laboratorio de Referencia de Escherichia coli (LREC), Departamento de Microbioloxía e Parasitoloxía, Facultade de Veterinaria, Universidade da Santiago de Compostela (USC), 27002 Lugo, Spain; 5 Department of Biochemistry and Molecular Biology, Faculty of Sciences, Campus Terra, Universidade da Santiago de Compostela (USC), 27002 Lugo, Spain

**Keywords:** *Escherichia coli*, chloramphenicol, multi-drug-resistant bacteria, extended-spectrum beta-lactamase, transposon-directed insertion site sequencing, antibiotic adjuvants

## Abstract

The increasing prevalence of antimicrobial resistance and the limited availability of new antimicrobial agents have created an urgent need for new approaches to combat these issues. One such approach involves reevaluating the use of old antibiotics to ensure their appropriate usage and maximize their effectiveness, as older antibiotics could help alleviate the burden on newer agents. An example of such an antibiotic is chloramphenicol (CHL), which is rarely used due to its hematological toxicity. In the current study, we employed a previously published transposon mutant library in MG1655/pTF2::*bla*_CTX-M-1_, containing over 315,000 unique transposon insertions, to identify the genetic factors that play an important role during growth in the presence of CHL. The list of conditionally essential genes, collectively referred to as the secondary resistome (SR), included 67 genes. To validate our findings, we conducted gene knockout experiments on six genes: *arcA*, *hfq*, *acrZ*, *cls*, *mdfA*, and *nlpI*. Deleting these genes resulted in increased susceptibility to CHL as demonstrated by MIC estimations and growth experiments, suggesting that targeting the products encoded from these genes may reduce the dose of CHL needed for treatment and hence reduce the toxicity associated with CHL treatment. Thus, the gene products are indicated as targets for antibiotic adjuvants to favor the use of CHL in modern medicine.

## 1. Introduction

Antimicrobial resistance (AMR) has emerged as a significant health issue, affecting both humans and animals and representing a substantial threat. According to estimates from 2019, bacterial AMR was associated with approximately 4.95 million deaths [1]. Within this figure, about 1.27 million deaths were directly attributed to bacterial AMR with *Escherichia coli*, *Staphylococcus aureus*, *Klebsiella pneumoniae*, *Streptococcus pneumoniae*, *Acinetobacter baumannii*, and *Pseudomonas aeruginosa* being the bacteria most linked to resistance with an estimation of 929,000 fatalities worldwide [1]. Hence, urgent action is necessary to tackle these antimicrobial-resistant bacteria.

One approach to address the problem of AMR involves revitalizing old and forgotten antibiotics that are not routinely used to treat bacterial infections due to their toxicity. Chloramphenicol (CHL), which is known for its bacteriostatic activity, is an example of an old and forgotten antibiotic. This antibiotic was originally obtained from *Streptomyces venezuelae* [2]. It interferes with protein synthesis by binding to peptidyl transferase, an enzyme that catalyzes the peptide bond formation at the 50S ribosomal subunit of 70S ribosomes [3]. Following its introduction, CHL became extensively utilized in both human and veterinary medicine [4,5]. However, the use of CHL has been widely reduced due to its association with hematological toxicity and incidences of serious side effects, such as aplastic anemia [4,6]. The reason behind these adverse drug reactions is assumed to be related to CHL’s interaction with mitochondrial ribosomes, which structurally resemble bacterial 70S ribosomes [5,6]. However, CHL is still employed in human medicine, though mainly in treating superficial bacterial infections like conjunctivitis and otitis externa. It is also utilized in treating a few life-threatening infections when no alternative antibiotics are available [7,8,9].

Extra-intestinal pathogenic *E. coli* (ExPEC) has recently been identified as the most common Gram-negative pathogen in humans [10]. Furthermore, the prevalence of extended-spectrum β-lactamase (ESBL)-producing ExPEC strains has increased significantly in recent years [10]. Since ExPEC strains are involved in a wide variety of extra-intestinal infections, including meningitis, urinary tract infections (UTIs), and septicemia [11], they have become a major challenge for clinical therapy. Thus, it is critical to combat these pathogens. CHL has been shown to be effective in treating a variety of infections, including meningitis and UTIs [5,12], due to its ability to be readily distributed to many body compartments and tissues. Besides antimicrobial resistance genes, a set of non-essential genes, unrelated to the resistance mechanism per se, is normally essential for the full expression of resistance. This set of genes has been denoted as the secondary resistome (SR) based on a study of genes affecting colistin resistance in *Klebsiella pneumoniae* [13]. The SR to CHL treatment in *E. coli* is currently unknown, and the aim of the current study was to determine this in a CTX-M-1-producing strain of *E. coli* during exposure to CHL at a ½ minimum inhibitory concentration (MIC). Additionally, this study aimed to explore whether deleting genes with a significant impact on fitness would improve the effectiveness of CHL, thereby enhancing treatment efficiency and safety.

## 2. Materials and Methods

### 2.1. Bacterial Strains and Antimicrobial Susceptibility Testing

The bacterial wild-type (WT) strain, mutant strains, and plasmids used in the current study are listed in Table 1. Difco™ lysogeny broth (LB) as well as Lennox (Becton, Dickinson, Albertslund, Denmark) and LB agar plates (Becton, Dickinson, Albertslund, Denmark) were used for bacterial growth. Bacterial strains were grown overnight at 37 °C except for the strains harboring the temperature-sensitive plasmid, pKD46, which were grown at 30 °C. The media were supplemented with 20 mg/L cefotaxime (CTX); 50 mg/L kanamycin (KAN); 20 mg/L gentamicin (GEN); and 1–50 mg/L CHL (Sigma, Copenhagen, Denmark) when appropriate. The MIC of CHL for the WT strain and mutants under study was determined via broth microdilution method following Clinical and Laboratory Standards Institute (CLSI)’s guidelines [14].

### 2.2. TraDIS Library, CHL Exposure, and Sequencing

A previously published transposon mutant library in MG1655/pTF2::*bla*_CTX-M-1_, containing over 315,000 unique transposon insertions [19], was employed to identify conditionally essential genes to CHL treatment. One ml aliquots of the input library carrying approximately 2 × 10^9^ mutants were prepared. From that, 100 μL was inoculated into a falcon tube containing 9.9 mL Mueller–Hinton Broth-II (MHB-II) (Becton, Dickinson, Albertslund, Denmark), supplemented with 2 mg/L, which corresponds to ½ MIC of CHL for MG1655/pTF2. Two biological replicates were performed. The genomic DNA of the output libraries was extracted after incubation at 37 °C for 24 h using GenElute™ Bacterial Genomic DNA kit (Sigma-Aldrich, Soeborg, Denmark) following manufacturer’s instructions. For qualification and quantification of DNA samples, ratios of 260/280 and 260/230 were measured using NanoDrop (Thermo Fisher Scientific, Roskilde, Denmark), and the concentrations were estimated using Qubit dsDNA HS Assay Kit (Thermo Fisher Scientific) (Table S1). To perform transposon-directed insertion site sequencing (TraDIS), DNA ranging from 2 to 4 μg was fragmented into approximately 300 bp fragments through mechanical shearing using Covaris M220 (Covaris, Woburn, MA, USA). Subsequently, each library was prepared for sequencing following the previously described protocols [20,21]. The quality and quantity of the PCR amplified fragments were evaluated via bioanalyzer (Agilent Technologies) and qPCR (Roche, Hvidovre, Denmark) [20,21]. The fragmented libraries were then pooled and sequenced on a MiSeq machine using a MiSeq reagent kit V2 (50 cycles) (Illumina) following the transposon sequencing recipe previously described [21].

### 2.3. Bioinformatic Analysis of TraDIS Data

The sequencing data were analyzed as previously described [19,20,21] using the Bio::TraDIS pipeline (https://github.com/sanger-pathogens/Bio-Tradis) (accessed on 27 June 2022). The generated files were then mapped to MG1655 reference genome (LR881938) using SMALT short-read mapper (https://www.sanger.ac.uk/tool/smalt-0/) (accessed on 27 June 2022), resulting in an accurate estimation of the insertion site of the transposon across the genome and unique insertion sites (UISs) (Table 2). The next analysis step was performed using the *tradis_comparisons.R* script, which revealed the log_2_ fold change (log_2_FC) of read counts and q value for each gene between the control without CHL and the test samples. The SR genes were defined as genes with a log_2_FC ≤ −2 and q value ≤ 0.01 for risk of false discovery. The full output data from R scripts showing the logFC and q value of each gene are described in Table S2. The raw sequence reads of this study were deposited in the European Nucleotide Archive (ENA) under the accession number PRJEB52919. STRING [22] analysis was used to determine the interactions between SR genes and to identify the enrichment of Kyoto Encyclopedia of Genes and Genomes (KEGG) terms and Gene Ontology (GO) terms based on the identified SR genes.

### 2.4. Construction of E. coli MG1655/pTF2 Mutant Strains

Mutant strains were made in MG1655/pTF2 for the genes *acrZ*, *cls* (synonymous with *clsA*), *mdfA*, *arcA*, *hfq*, and *nlpI* using the Lambda Red recombination system essentially as described [17,18]. Deletion mutants were confirmed via PCR. The primers used for creating and confirming mutants are listed in Table S3.

### 2.5. Construction of Plasmid pACYC184_Backbone Lacking TET^R^

The pACYC184-CHL harboring a CHL resistance gene (*cat*, which encodes a CHL acetyltransferase) was generated from pACYC184 as template (GenBank X06403) using HiFi cloning (New England BioLabs, Ipswich, MA, USA) and PCR. The PCR fragment for cloning was amplified with primers listed in Table S3 using Q5 DNA polymerase (NEB). The purified HiFi cloning product (pACYC184-CHL) was used to transform electrocompetent *E. coli* DH5α. The recombinant plasmid was extracted using GeneJET Plasmid Miniprep (Thermo Fisher Scientific, Roskilde, Denmark) and confirmed by Sanger sequencing (Macrogen Europe). Then, MG1655/pTF2 and mutants were transformed with the recombinant plasmid using electroporation.

### 2.6. Growth Experiments

To validate the predictions revealed by the TraDIS analysis, the WT strain (MG1655/pTF2) and its mutant derivatives (Δ*acrZ*, Δ*cls*, Δ*mdfA*, Δ*arcA*, Δ*hfq*, and Δ*nlpI*) were compared in terms of their ability to grow without antibiotics and in the presence of CHL using Bioscreen C (Thermo Labsystems, Helsinki, Finland) as previously described with slight modifications [19]. Briefly, a 10^−2^ dilution of 0.5 MacFarland (1–2 × 10^8^ CFU/mL) was prepared in MHB-II for each strain, resulting in a final cell density of approximately 10^6^ CFU/mL. Then, 250 μL of the bacterial suspension was inoculated in each well. Next, the cultures were grown without antibiotics and in the presence of 2 mg/L CHL. The OD_600_ was measured every 30 min with continuous shaking for 24 h at 37 °C. This experiment was performed with two biological replicates using two technical replicates, and the growth curves were generated using GraphPad Prism 9 (GraphPad Software, San Diego, CA, USA). 

### 2.7. Homology to Human Proteins

To determine whether the potential targets, which displayed increased susceptibility to CHL upon deletion, have similarities to the human proteome, the protein sequences of ArcA, Hfq, AcrZ, CLS, MdfA, and NlpI were retrieved from Artemis. These sequences were then subjected to a sequence homology search with human proteins using the NCBI Homo sapiens Protein BLAST (BLASTp) tool, accessible at http://blast.ncbi.nlm.nih.gov/Blast.cgi (accessed on 20 February 2023). Proteins that did not yield any matches with an E-value cutoff of 10^−10^ were identified as non-homologous proteins [23].

## 3. Results

### 3.1. MIC Testing of MG1655/pTF2

In our previous study, we reported that MG1655/pTF2 displayed resistance to CTX, with an MIC value of 256 mg/L [19]. This resistance is attributed to the presence of *bla_CTX-M-1_* in the IncI1 plasmid. In the current study, we assessed the MIC of CHL for the same strain. The results showed that MG1655/pTF2 exhibited an MIC value of 4 mg/L for CHL. Based on the CLSI and the European Committee on Antimicrobial Susceptibility Testing (EUCAST) breakpoints [14,24], MG1655/pTF2 is considered to be susceptible to CHL.

### 3.2. Uncovering the SR Genes to CHL in MG1655/pTF2

To identify the SR genes during the exposure to CHL, transposon insertions were compared between the control output libraries (input libraries grown in the absence of antibiotics), which had been previously sequenced and analyzed [19], and the input libraries grown in the presence of a ½ MIC of CHL. The bioinformatic analysis reveals that 67 genes were identified as SR genes to CHL (Table S2).

The top 20 significantly affected/disrupted genes during growth of the bacteria in the presence of CHL, encompassing the genes selected for validation studies, are listed in Table 3. The gene *nlpI*, encoding the lipoprotein NlpI, which co-ordinates peptidoglycan synthesis and hydrolysis [25], was predicted to yield the highest fitness defect (Log_2_FC = −6.45), followed by *acrZ*, encoding an assessor protein in a multidrug efflux pump [26] (Log_2_FC = −5.41). 

A visual representation of the interactions between the genes that were significantly affected during growth in the presence of a ½ MIC of CHL is shown in Figure S1. Our analysis revealed the enrichment of only one KEGG pathway (cation antimicrobial peptide (CAMP) resistance). Five GO terms were enriched in the biological processes category, including lipid transport, membrane organization, intermembrane lipid transfer, Gram-negative-bacterium-type cell outer membrane assembly, and biological regulation. No terms were enriched in the molecular functions category, while the terms protein containing complex, efflux pump complex, and cell envelope were enriched in the cellular components category (Table S4).

The genes highlighted in Table 3 were chosen for further validation through site-specific deletions, followed by an assessment of their growth performance in the absence and presence of CHL and an MIC analysis.

### 3.3. Validation of SR Genes to CHL

Based on the analyses performed above, *acrZ*, *cls*, *mdfA*, *arcA*, *hfq*, and *nlpI* were knocked out in MG1655/pFT2 by site-specific deletion to investigate their importance for growth in the absence and presence of CHL and to examine their effect on the MIC. These genes are associated with multidrug efflux pumps (*acrZ*, *mdfA*) [26,27], cardiolipin biosynthesis (*cls*) [28], transcriptional and post-transcriptional regulation (*arcA*, *hfq*) [29,30], and cell division (*nlpI*) [31].

**Figure 1:**
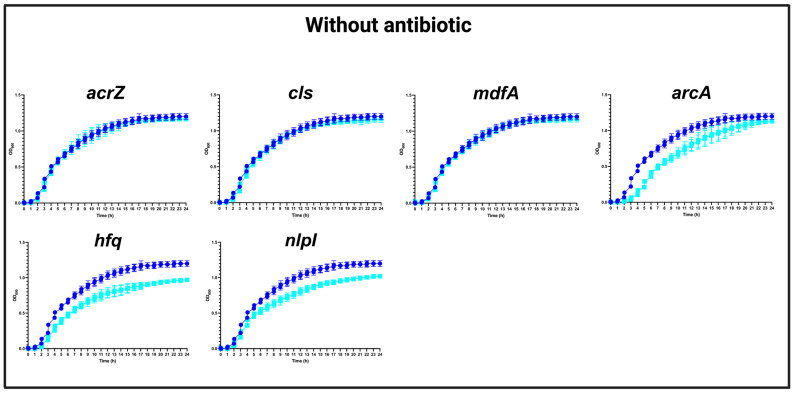
Growth curves of MG1655/pTF2 (WT) and *acrZ*, *cls*, *mdfA*, *arcA*, *hfq*, and *nlpI* mutants in the absence of antibiotics. Growth curves of WT (blue line) against mutants (cyan lines) in MHB-II without CHL. The data shown are means ± standard deviations of two biological replicates with two technical replicates.

In the absence of CHL, our analysis revealed that the mutations in *acrZ*, *cls*, and *mdfA* did not affect the growth compared to the WT strain. However, mutations in *arcA*, *hfq*, and *nlpI* yielded a slight growth defect (Figure 1), which should be considered in the interpretation of the results below.

**Figure 2:**
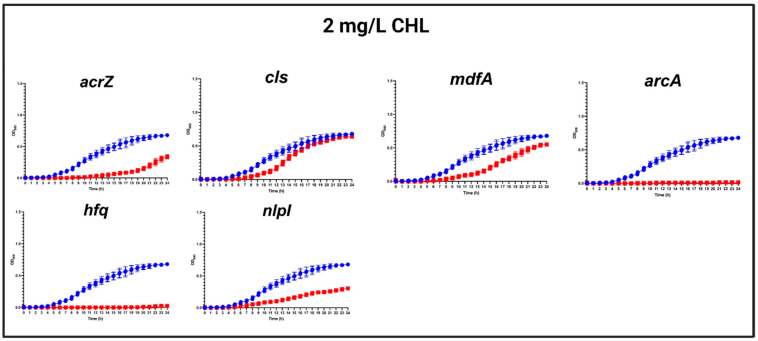
Growth curves of MG1655/pTF2 (WT) and *acrZ*, *cls*, *mdfA*, *arcA*, *hfq*, and *nlpI* mutants in the presence of 2 mg/L CHL. Growth curves of WT (blue line) against mutants (red lines) in MHB-II in the presence of CHL. The data shown are means ± standard deviations of two biological replicates with two technical replicates.

In the presence of a ½ MIC (2 mg/L) of CHL, the most obvious growth defects were found in mutants with a specific deletion of *arcA*, *hfq*, *acrZ*, and *nlpI*, respectively, while a slighter growth defect was observed for Δ*mdfA*, and almost no difference in growth was detected for Δ*cls* compared to the WT strain (Figure 2).

### 3.4. Improved Efficacy to CHL

To further explain the growth experiments mentioned above, we analyzed the MICs of CHL for the selected mutant strains. The mutations in *arcA* and *hfq* led to a four-fold reduction in the MIC of CHL, whereas the mutations in *acrZ*, *cls*, *mdfA,* and *nlpI* led to a two-fold reduction in the MIC of CHL (Table 4).

Since MG1655/pTF2 is sensitive to CHL with an MIC of 4 mg/L, we extended our analysis to investigate if these findings are applicable to CHL-resistant strains. Consequently, the plasmid pACYC184-CHL harboring the *cat* gene that was constructed in the present study was transformed into the WT and mutant strains. Based on our susceptibility testing, a similar decreasing trend was documented in the MIC of CHL in the CHL-resistant mutant strains compared to the CHL-sensitive mutant strains (Table 4).

### 3.5. Homology of Proteins Encoded by the Selected Genes to Human Proteins

To assess the potential suitability of the identified targets for the development of antibiotic adjuvants, the protein sequences of ArcA, Hfq, AcrZ, CLS, MdfA, and NlpI were compared to the human proteome using NCBI-BLAST tools. This analysis aimed to minimize any undesired side effects on the host. The in silico analysis did not reveal any similarity between the ArcA, Hfq, AcrZ, CLS, MdfA, and NlpI proteins from MG1655/pTF2 and human proteins.

## 4. Discussion

AMR has become a pressing issue in the healthcare sector, affecting individuals worldwide. As a result, AMR has been recognized by the World Health Organization (WHO) as a major threat to global health security [32]. The growing prevalence of infections caused by multidrug-resistant Gram-negative bacilli (MDR-GNB) poses a serious challenge in healthcare facilities due to the lack of effective treatment options [33]. Additionally, no new antibacterial agents with activity exclusively against Gram-negative bacteria or resistant bacteria that withstand currently available antibiotics are currently being developed [34]. Due to this, there is a renewed interest in reconsidering older and forgotten antibiotics that have been largely abandoned due to their toxic side effects. One such antibiotic is CHL, which was extensively used in 1950s [35]. CHL’s popularity waned in the 1960s due to its toxicity, which caused two types of bone marrow effects: dose-dependent reversible anemia and unpredictable, irreversible, frequently fatal aplastic anemia. The occurrence rate of aplastic anemia was estimated to be 1 case in 24,000 to 40,000 therapy courses [36]. The rationale for reconsidering CHL is strengthened by its limited recent usage, which reduces the opportunity for bacteria to develop resistance against it [33]. Also, CHL is a broad-spectrum antibiotic that displays a powerful activity against diverse types of microorganisms, including Gram-positive and Gram-negative bacteria [37]. In addition to its ability to penetrate different tissues, CHL can be administered by several different routes, including oral, parenteral, or topical routes. Moreover, CHL is considered an inexpensive antibiotic and has been recommended by the WHO in circumstances in which modern alternatives are scarce, which is a frequently encountered scenario in low-income countries [37,38,39]. Hence, this study employed a high-throughput TraDIS screening method to identify the genes, other than the resistance genes, necessary for the growth of *E. coli* when exposed to CHL. Our analysis showed that a total of 67 genes were significantly affected by transposon insertions upon CHL exposure, representing the SR to CHL in MG1655/pTF2 *E. coli*. Therefore, their encoded products might be considered as target(s) for antibiotic adjuvants which, when applied in combination with CHL, may enhance the efficacy of antibiotics.

The SR to CHL has not previously been characterized. The pathway analysis in the CHL-sensitive strain under study via KEGG showed enrichment of only one pathway: resistance to antimicrobial peptides. The biological reason for this was not investigated further, but the genes involved included *acrB*, *acrA*, *phoQ*, *phoP*, *marA*, and *tolC*, which are related to two-component systems involved in stress response and to efflux pumps [40,41,42,43]. This suggests that efflux may play an important role in tolerating CHL in this strain. Our analysis of GO terms in different categories, however, suggested that a broader set of biological processes and cellular components (lipid transport, membrane organization, intermembrane lipid transfer, outer membrane assembly, protein-containing complex, and cell envelope) were important for growth in the presence of CHL. It remains to be investigated whether this is also the case in CHL-resistant *E. coli*.

To validate our results, we knocked out six genes that are linked to multidrug efflux pumps (*acrZ*, *mdfA*) [26,27], cardiolipin biosynthesis (*cls*) [28], transcriptional and post-transcriptional regulation (*arcA*, *hfq*) [29,30], and cell division (*nlpI*) [31]. The deletion of these genes in MG1655/pTF2 was found to increase sensitivity to CHL both in the original CHL-sensitive strain and in the derived CHL-resistant mutants, suggesting that the SR genes to CHL might be shared between sensitive and resistant strains. Moreover, our results agreed with those of previous studies showing that *acrZ* and *mdfA* are important for the efflux of CHL [26,44], which suggests the accuracy and reliability of the TraDIS approach used in this study. The other genes identified here (*cls*, *hfq*, *nlpI*, and *arcA*) have not previously been shown to play a role in resistance to CHL.

The *cls* gene encodes a cardiolipin synthase categorized as a non-essential protein for growth in many culture conditions [28,45], and in agreement with this, the mutant was not affected in terms of growth in the absence of antimicrobials. The deletion of *cls* was previously found to increase susceptibility to novobiocin [46], which, together with the increased susceptibility to CHL detected in our study, suggests that it may represent a target for an antibiotic adjuvant for several antimicrobials.

Our TraDIS analysis showed that *hfq* and *nlpI* were also important for growth of MG1655/pTF2 in the presence of CHL. Additionally, these genes have been previously proven to be relevant for growth when exposed to CTX [19]. Thus, as mentioned for *cls*, *hfq* and *nlpI* might be investigated as potential targets for different antimicrobials. Based on the log_2_FC, however, both genes might be more important for growth in the presence of CHL than in the presence of CTX. The deletion of *nlpI* was previously found to be associated with the production of high levels of MepS during growth of the bacteria [25] and the increased production of outer membrane vesicles (OMVs) [47,48]. This hypervesiculating phenotype, which is associated with improved *E. coli* survival in the presence of β-lactam antibiotics [49], was suggested to be the result of the high levels of peptidoglycan synthesis and the low-level production of lipoprotein outer membranes. Since the deletion of *mepS* was previously observed to suppress the high production of OMVs associated with the deletion of *nlpI* [50], we hypothesize that the *nlpI* mutant may exhibit a decreased susceptibility to CTX due to its association with the hypervesiculating phenotype; however, further studies are needed to confirm this hypothesis.

Among the genes we validated, the deletion of *arcA* led to a four-fold reduction in the MIC of CHL in MG1655/pTF2. *arcA* encodes the response regulator of the two-component system ArcA/B, which is a global regulator of gene expression under anaerobic and microaerobic conditions [51], and under anaerobic conditions, this system is known to repress the protein expression involved in aerobic respiration [52]. The deletion of *arcA* has been previously reported to be associated with activating aerobic respiration in anaerobic and microaerobic conditions [53], resulting in the enhancement of the reactive oxygen species (ROS) production in *E. coli* BW25113 [54]. Furthermore, it was demonstrated that the deletion of *arcA* significantly suppresses the evolution of resistance to ciprofloxacin, cefixime, and CHL [54], and the expression of *arcA* was found to be increased in *E. coli* strains resistant to quinolones, β-lactams, and CHL [54,55]. Since ArcA is not an essential protein [56], and several studies have proposed that ROS production contributes to antibiotic-mediated cell killing [57,58], we suggest that *arcA* could be a potential target to enhance the efficacy of CHL to treat infections caused by *E. coli*. 

Unlike the development of conventional antibiotics, developing antibiotic adjuvants has the advantage of not requiring the identification of a target that is essential for bacterial survival, but a target, that, when inactivated, improves the potency of conventional antibiotics [59]. Nonetheless, there are several essential safety considerations that must be taken into account to consider a target as a promising candidate for antibiotic adjuvant development. For instance, the target should have minimal or no antibacterial activity when blocked, and it should not have similarities to host proteins [13]. These characteristics are crucial in reducing the likelihood of resistance development against antibiotic adjuvants and minimizing any undesirable effects on the host. In this context, our analysis of the growth experiments revealed no significant impacts on growth when *acrZ*, *cls*, and *mdfA* were inactivated compared to the WT strain in the absence of CHL. On the other hand, deletions of *arcA*, *hfq*, and *nlpI* resulted in a slight growth defect under similar conditions. This suggests that blocking these genes has limited or no antibacterial effects against the bacteria. Additionally, no significant similarities between AcrZ, CLS, MdfA, ArcA, Hfq, NlpI, and human proteins were detected. Taken together, these genes could serve as potential targets for antibiotic adjuvants to enhance the effectiveness of CHL, enabling treatment with lower doses and minimizing its toxic side effects. However, caution is needed when extrapolating the findings of this study, because they were based on a single *E. coli* strain, MG1655, which is non-pathogenic. Therefore, the possibility of using the identified genes as antibiotic adjuvant targets needs to be further validated by evaluating the effect of their deletion in pathogenic *E. coli* strains. This would also allow for the testing of the effect of the deletion of genes on the treatment efficacy of CHL against resistant strains in animal models by a comparison of infection outcomes following the challenge and treatment of wild-type and gene knockout mutants. 

The small RNA molecule, *arcA*, is involved in the control of chemotaxis and motility in *E. coli*, and it has been shown to influence virulence in avian pathogenic *E. coli* [60]. Additionally, cardiolipin, whose synthesis requires *cls*, is important for virulence in *Shigella flexnerii*, a close relative of *E. coli* [61]. Thus, blocking these genes may have additional effects, reducing resistance to CHL, and further studies into the effects of blocking all the genes we have selected on virulence are warranted. Further, as already discussed for some of the genes, there may be effects on the susceptibility to other antibiotics than CHL. Thus, the deletion of *hfq* has pleiotropic effects because it encodes a chaperone, which assists several small RNA molecules when they exert their effects on gene expression [62]. A study has shown that the deletion of this gene affects the susceptibility to antibiotics of several classes [63]. Similarly, MFS efflux pumps, to which MdfA belongs, can export several drug classes and are present in many bacteria [64]. Thus, further studies on the possible effects of blocking these genes in bacteria other than *E. coli* are highly relevant.

In conclusion, this study provided the first list of genes that are fitness genes for growth in the presence of CHL in an *E. coli*-sensitive strain. Moreover, the deletion of the six selected genes increased the sensitivity to CHL, not only in the WT sensitive strain but also in the derived mutants resistant to CHL. Such genes could potentially serve as targets for antibiotic adjuvants, enabling more effective treatments of MDR bacteria with lower CHL concentrations compared to using CHL alone and therefore yielding a lower level of toxicity. Besides CHL, some of these might be helper drugs for other antibiotics. 

## Figures and Tables

**Table 1:** *E. coli* strains and plasmids used in this study.

Strain	Genotype	Reference
MG1655/pTF2	*E. coli MG1655 + bla_CTX-M-1_ on IncI1 plasmid pTF2*	[15]
ATCC^®^ 25922	*E. coli* Reference strain	[16]
JEO-6259	MG1655 Δ*acrZ* (KAN^R^)/pTF2 (CTX^R^)	This study
JEO-6260	MG1655 Δ*cls* (KAN^R^)/pTF2 (CTX^R^)	This study
JEO-6261	MG1655 Δ*mdfA* (KAN^R^)/pTF2 (CTX^R^)	This study
JEO-6262	MG1655 Δ*arcA* (KAN^R^)/pTF2 (CTX^R^)	This study
JEO-6263	MG1655 Δ*hfq* (KAN^R^)/pTF2 (CTX^R^)	This study
JEO-6264	MG1655 Δ*nlpI* (KAN^R^)/pTF2 (CTX^R^)	This study
MSA-001	MG1655/pTF2 (CTX^R^)/pACYC184_ΔTET^R^ (CHL^R^)	This study
JEO-6265	MG1655 Δ*acrZ* (KAN^R^)/pTF2 (CTX^R^)/pACYC184_ΔTET^R^ (CHL^R^)	This study
JEO-6266	MG1655 Δ*cls* (KAN^R^)/pTF2 (CTX^R^)/pACYC184_ΔTET^R^ (CHL^R^)	This study
JEO-6267	MG1655 Δ*mdfA* (KAN^R^)/pTF2 (CTX^R^)/pACYC184_ΔTET^R^ (CHL^R^)	This study
JEO-6268	MG1655 Δ*arcA* (KAN^R^)/pTF2 (CTX^R^)/pACYC184_ΔTET^R^ (CHL^R^)	This study
JEO-6269	MG1655 Δ*hfq* (KAN^R^)/pTF2 (CTX^R^)/pACYC184_ΔTET^R^ (CHL^R^)	This study
JEO-6270	MG1655 Δ*nlpI* (KAN^R^)/pTF2 (CTX^R^)/pACYC184_ΔTET^R^ (CHL^R^)	This study
Plasmids		
pKD4	rep_R6K_ γAMP^R^ FRT KAN^R^ FRT	[17]
pKD46	rep_pSC101_^ts^ GEN^R^ P*_araBAD_*γβ *exo*	[18]
pACYC184_ΔTet^R^	pACYC184 lacking TET^R^	This study

KAN^R^, CTX^R^, CHL^R^, AMP^R^, GEN^R^, and TET^R^: kanamycin-, cefotaxime-, chloramphenicol-, ampicillin-, gentamicin-, and tetracycline-resistant, respectively.

**Table 2:** Mapping and read counts of Tn5 insertions to K-12 MG1655 reference genome.

Library	Total Reads	Reads Mapped (%) ^1^	Total UIS ^2^	Total Seq Length/Total UIS
Input				
MG1655_pTF2_input_1 ^3^	10,795,077	90.79	234,898	19.69
MG1655_pTF2_input_2 ^3^	11,001,585	85.73	279,308	16.56
Input 1 + Input 2 (combined)^3^	21,796,662	88.23	315,925	14.64
Output				
MG1655_pTF2_without_antibiotic_1 ^3^	11,238,753	89.03	229,617	20.14
MG1655_pTF2_without_antibiotic_2 ^3^	12,879,322	89.20	233,968	19.77
MG1655_pTF2_2mg_CHL_1	13,521,046	93.21	126,609	36.53
MG1655_pTF2_2mg_CHL_2	9,064,880	85.44	240,182	19.26

^1^ Percentage of mapped sequence reads to K-12 MG1655 reference genome, ^2^ unique insertion sites, and ^3^ libraries that have been previously sequenced and analyzed [19].

**Table 3:** Top twenty affected genes in the SR to CHL in MG1655/pTF2.

Gene	Function	2 mg/L CHL	
		Log_2_FC	q. Value
*nlpI* ^1^	lipoprotein NlpI	−6.24	2.10 × 10^−13^
*acrZ* ^1^	multidrug efflux pump accessory protein AcrZ	−5.41	1.03 × 10^−11^
*pbgA*	cardiolipin transport protein PbgA	−5.40	1.54 × 10^−10^
*tolC*	outer membrane channel protein TolC	−5.39	1.00 × 10^−11^
*lptC*	LPS export ABC transporter periplasmic protein LptC	−5.33	1.30 × 10^−5^
*prc*	carboxy terminal-processing peptidase	−5.10	6.93 × 10^−8^
*acrA*	multidrug efflux RND transporter periplasmic adaptor subunit AcrA	−5.01	1.56 × 10^−10^
*acrB*	multidrug efflux RND transporter permease subunit	−4.97	3.87 × 10^−7^
*lptA*	lipopolysaccharide ABC transporter substrate-binding protein LptA	−4.75	3.87 × 10^−5^
*mdfA* ^1^	multidrug efflux MFS transporter MdfA	−4.58	1.23 × 10^−20^
*cls* ^1^	cardiolipin synthase	−4.23	5.42 × 10^−9^
*lpp*	murein lipoprotein Lpp	−4.15	0.0002
*phoQ*	two-component system sensor histidine kinase PhoQ	−4.08	5.14 × 10^−6^
*Hfq* ^1^	RNA chaperone Hfq	−4.08	1.00 × 10^−8^
*arcA* ^1^	two-component system response regulator ArcA	−4.07	5.14 × 10^−6^
*qseC*	two-component system sensor histidine kinase QseC	−3.98	5.84 × 10^−8^
*prmB*	50S ribosomal protein L3 N(5)-glutamine methyltransferase	−3.96	0.0001
*hrpA*	ATP-dependent RNA helicase HrpA	−3.92	8.75 × 10^−5^
*ihfA*	integration host factor subunit alpha	−3.88	0.0049
*rpoS*	RNA polymerase sigma factor RpoS	−3.73	2.24 × 10^−9^

^1^ Indicates genes which were selected for mutagenesis in relation to validation study.

**Table 4:** MICs of CHL against MG1655 mutants.

Strain	CHL (mg/L)
**MG1655/pTF2 (WT)**	4
Δ*acrZ*	**2**
Δ*cls*	**2**
Δ*mdfA*	**2**
Δ*arcA*	**1**
Δ*hfq*	**1**
Δ*nlpI*	**2**
**MG1655/pTF2/pACYC184-CHL/ΔTetR**	1024
Δ*acrZ*	**512**
Δ*cls*	**512**
Δ*mdfA*	**512**
Δ*arcA*	**256**
Δ*hfq*	**256**
Δ*nlpI*	**512**

Bold MIC values indicate a reduction in MIC compared to the WT strain.

## Data Availability

The data presented in this study are available in the article and Supplementary Materials.

## Supplementary Materials

The following supporting information can be downloaded at https://www.mdpi.com/article/10.3390/antibiotics13010073/s1. Table S1: DNA quality and quantity of CHL-treated libraries; Table S2: The full output from R scripts; Table S3: Primers used in mutant strains and plasmid pACYC184 backbone construction; Table S4: KEGG pathway and GO terms enrichment analysis for genes identified as SR to CHL; Figure S1: Representation of the interactions between the genes identified as SR to CHL.

## Abbreviations

CHL	Chloramphenicol
SR	Secondary resistome
MIC	Minimum inhibitory concentration
AMR	Antimicrobial resistance
ExPEC	Extra-intestinal pathogenic *E. coli*
ESBL	Extended-spectrum β-Lactamase
UTIs	Urinary tract infections
CTX-M	Cefotaximase from Munich
WT	Wild-type
LB	Lysogeny broth
CTX	Cefotaxime
KAN	Kanamycin
GEN	Gentamicin
AMP	Ampicillin
TET	Tetracycline
MHB-II	Mueller–Hinton Broth-II
TraDIS	Transposon-directed insertion site sequencing
PCR/qPCR	Polymerase Chain Reaction/Quantitative Polymerase Chain Reaction
UIS	Unique insertion sites
ENA	European Nucleotide Archive
STRING	Search Tool for the Retrieval of Interacting Genes/Proteins
KEGG	Kyoto Encyclopedia of Genes and Genomes
GO	Gene Ontology
CAT	Chloramphenicol acetyltransferase
CFU	Colony forming unit
NCBI	National Center for Biotechnology Information
BLAST	Basic Local Alignment Search Tool
CLSI	Clinical and Laboratory Standards Institute
EUCAST	European Committee on Antimicrobial Susceptibility Testing
CAMP	Cation Antimicrobial Peptide
WHO	World Health Organization
MDR	Multidrug resistance
GNB	Gram-negative bacilli
OMV	Outer membrane vesicles
ROS	Reactive oxygen species
