# Supplementary material for: Enhancing the Efficacy of Chloramphenicol Therapy for Escherichia coli by Targeting the Secondary Resistome
Source: Antibiotics (Basel). 2024 Jan 12;13(1):73. doi: 10.3390/antibiotics13010073 (PMC10812820; doi:10.3390/antibiotics13010073)
Supplement: Supplementary file 1 [file antibiotics-13-00073-s001.zip › Figures S1.pdf]

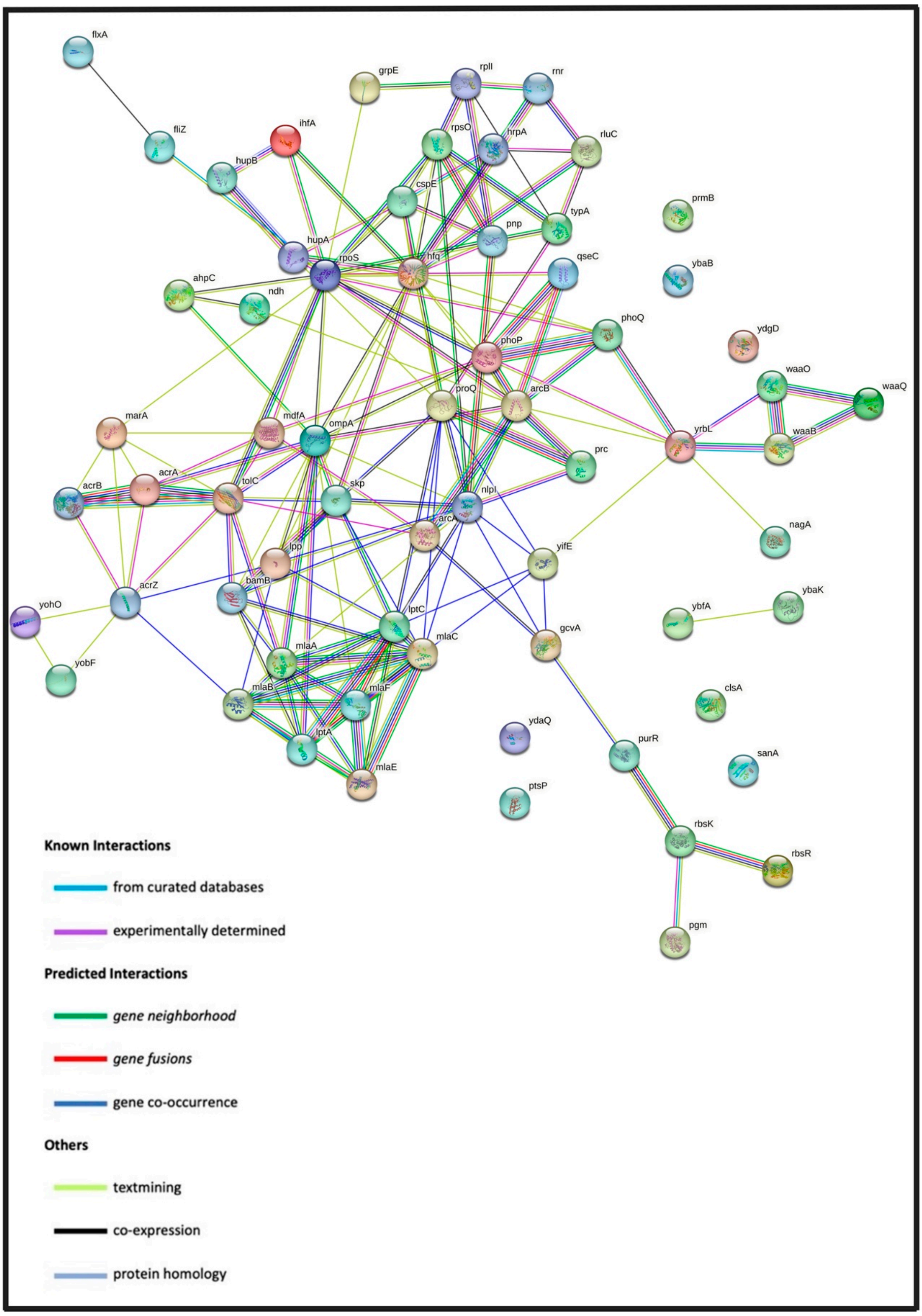

Figure S1. STRING analysis visual representation of the interactions between the genes identified as SR to CHL treatment.
