# Supplementary material for: Enhancing the Efficacy of Chloramphenicol Therapy for Escherichia coli by Targeting the Secondary Resistome
Source: Antibiotics (Basel). 2024 Jan 12;13(1):73. doi: 10.3390/antibiotics13010073 (PMC10812820; doi:10.3390/antibiotics13010073)
Supplement: Supplementary file 1 [file antibiotics-13-00073-s001.zip › Tables S1_S3.pdf]

Table S1. DNA quality and quantity of CHL-treated libraries.

Condition	T0 CFU/ml	T24 CFU/ml	260/280 (Nanodrop)	260/230 (Nanodrop)	Qbit HS[1] ng/µl
MG1655/pFT2_2µg_CHL_1	3×10^6	1×10^9	1.87	2.15	124
MG1655/pFT2_2µg_CHL_2	1.6×10^6	7.5×10^8	1.84	2	184

[1]dsDNA HS (High Sensitivity) Assay Kit.

Table S3. Primers used for mutants creation, verification, construction of plasmid pACYC184 backbone.

Primers[1]	Sequence (5′-3′)
acrZ-F	AGCGGTTAGAATAGTCTCATGACTATATCTGGAGTTGACCTGTAGGCTGGAGCTGCTTC
acrZ-R	GAGAAAAAGAAAATGCCCGACTAAGCGGGCATTCAGGGAACATATGAATATCCTCCTTAG
cls-F	TAAACTCATAACAATGCGCTTTCAAAAGGATTTCTAATCTTGTAGGCTGGAGCTGCTTC
cls-R	ATCCATAGTTACTACCTGTTTAACCTCTGTTGGCGACGTTCATATGAATATCCTCCTTAG
mdfA-F	TTATTAAACTCTGCGCGATTATTATTGGCGAAGAAATTGCTGTAGGCTGGAGCTGCTTC
mdfA-R	TTCGAGAATGCCTGATCGCACAAAGCAGTCAGGCATTTTTCATATGAATATCCTCCTTAG
arcA-F	ACTTCCTGTTTCGATTTAGTTGGCAATTTAGGTAGCAAACTGTAGGCTGGAGCTGCTTC
arcA-R	CGCTAAAAAGCGCCGTTTTTTTTGACGGTGGTAAAGCCGACATATGAATATCCTCCTTAG
hfq-F	AAGGTTCAAAGTACAAATAAGCATATAAGGAAAAGAGAGATGTAGGCTGGAGCTGCTTC
hfq-R	AGGATCGCTGGCTCCCCGTGTAAAAAAACAGCCCGAAACCCATATGAATATCCTCCTTAG
nlpI-F	AACAGGACGTTCATTCAACCGTGGTCTTCGGGAGTGGGAATGTAGGCTGGAGCTGCTTC
nlpI-R	ATGGCAATCAAAAAAGATTACGGGCTGATGTGTACGTCAGCATATGAATATCCTCCTTAG
**Primers[2]**	**Sequence (5′-3′)**
acrZ-F	CTCCCTGGCTAATGGAACCG
acrZ-R	TCTGCCAGTGCATTGCCAGC
cls-F	ATATTTCGGCGCGATTGCCC
cls-R	AAGTCCGGGTTCAAATCGAA
mdfA-F	GTCGTTGAGAATAAAGTGCA
mdfA-R	TGCCGAGTGGATACAAAGTG
arcA-F	CCTGTTATGTTGCTGTTAAA
arcA-R	GATGTGGGGACTGCTCAACT
hfq-F	AAATGGTTTATCGAGGTGTT
hfq-R	CTTTGGGTGCGGCGCTTTAC
nlpI-F	GGTCAGGAAGTACCGGTGAA
nlpI-R	GGGCTTCAAGGATAGGAGCC
**Primers[3]**	**Sequence (5′-3′)**
pACYC184_F	ATTCTCATGTGAGTCAACGCCATGAGC
pACYC184_R	GCGTTGACTCACATGAGAATTACAACTTATATCGTATG

[1]Primers used for mutant creation.

[2]Primers used for proof of insertion.

[3]Primers used for pACYC184 backbone construction.
